# The NASA ABoVE Land, Vegetation, and Ice Sensor full waveform LiDAR airborne surveys

**DOI:** 10.1038/s41597-025-06388-5

**Published:** 2026-07-28

**Authors:** Elizabeth E. Hoy, Charles E. Miller, Peter C. Griffith, Paul M. Montesano, Michelle Hofton, Sandra Yaacoub, J. Bryan Blair, Amanda Armstrong, Colleen Brooks, Helen Cornejo, Laura Duncanson, Scott J. Goetz, Shane Hendry, Daniel Hopf, Michael E. Howerton, Lisa Kaser, John S. Kimball, Libby Larson, Matthew J. Macander, Matthew Mullin, David Rabine, Laurence C. Smith, Sarah Story, Robert Switzer, Hana Travers-Smith, Jurjen Van der Sluijs, Yaxing Wei, Albert Wu, Daryl Yang

**Affiliations:** 1 NASA Goddard Space Flight Center, Greenbelt, Maryland 20771 USA; 2 Global Science & Technology, LLC, Greenbelt, Maryland 20770 USA; 3 Jet Propulsion Laboratory, California Institute of Technology, Pasadena, California 91109 USA; 4 Science Systems and Applications Inc., Lanham, Maryland 20706 USA; 5 ADNET Systems Inc., Bethesda, Maryland 20817 USA; 6 Department of Geographical Sciences, University of Maryland, College Park, Maryland 20742 USA; 7 Department of Geography and Planning, Queen’s University, Kingston, Ontario K7L 3N6 Canada; 8 Earth System Science Interdisciplinary Center - University of Maryland, College Park, Maryland 20742 USA; 9 KBR, Fulton, Maryland 20770 USA; 10 School of Informatics and Computing, Northern Arizona University, Flagstaff, Arizona 86011 USA; 11 National Snow and Ice Data Center Distributed Active Archive Center, Cooperative Institute for Research in Environmental Sciences, University of Colorado, Boulder, Colorado 80303 USA; 12 Numerical Terradynamic Simulation Group, University of Montana, Missoula, Montana 59812 USA; 13 ABR, Inc., Fairbanks, Alaska 99709 USA; 14 Department of Earth, Environmental and Planetary Sciences and Institute at Brown for Environment and Society, Brown University, Providence, Rhode Island 02912 USA; 15 Faculty of Forestry, University of British Columbia, Vancouver, British Columbia V6T 1Z4 Canada; 16 NWT Centre for Geomatics, Government of the Northwest Territories, Yellowknife, Northwest Territories X1A K3 Canada; 17 Environmental Sciences Division, Oak Ridge National Laboratory, Oak Ridge, Tennessee 37830 USA

**Keywords:** Carbon cycle, Boreal ecology, Forest ecology, Ecosystem ecology

## Abstract

Arctic and boreal regions are experiencing rapid environmental changes that include thawing permafrost and increasing disturbances. The NASA Arctic-Boreal Vulnerability Experiment (ABoVE) sought to better understand these changes through field, airborne, and remote sensing measurements. One key airborne instrument was the Land, Vegetation, and Ice Sensor (LVIS), a wide-swath imaging laser altimeter system. LVIS conducted 32 flights during June-August periods of 2017 and 2019, capturing data across more than 91,000 km² of diverse Arctic and boreal ecosystems. The surface topography and vegetation structure data collected throughout Alaska and Northwestern Canada spans boreal forests to Arctic tundra, crossing 12 distinct ecoregions. This airborne collection enables direct comparison with coincident NASA Ice, Cloud, and Land Elevation Satellite-2 (ICESat-2) data, extends research beyond the ~52° N limit of NASA’s Global Ecosystem Dynamics Investigation (GEDI) sensor, and provides precursor data for future satellite missions, such as NASA’s recently selected Earth Dynamics Geodetic Explorer (EDGE). We summarize detailed information on LVIS data records from ABoVE deployments, including access and visualization using custom open source tools.

## Background & Summary

**Fig. 1:**
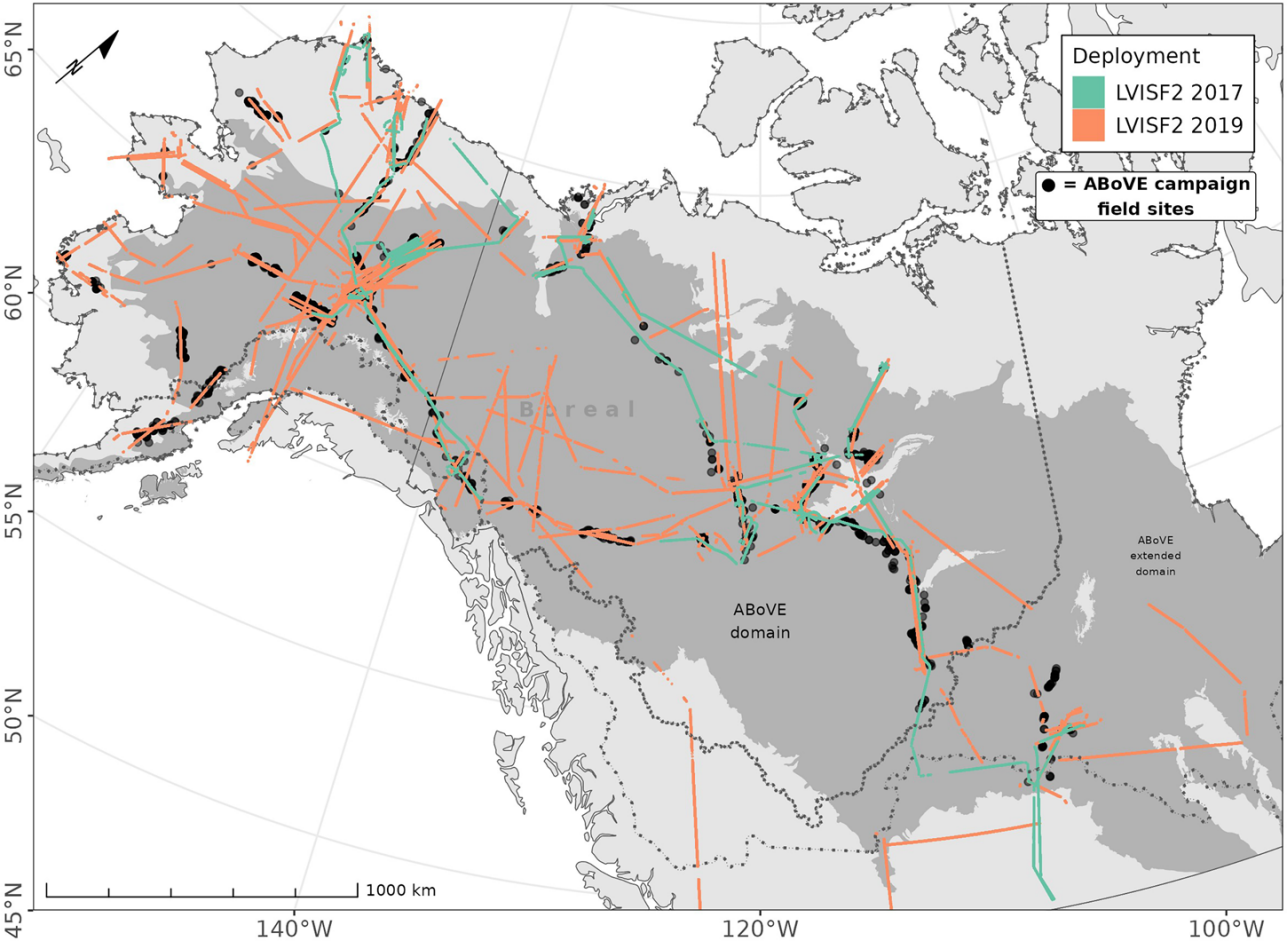
Map of the 2017 and 2019 LVIS deployments for ABoVE. The ABoVE LVIS Level-3 flight line footprints from Montesano *et al*.^76^ are shown in green (2017) and orange (2019), field sites used in developing the ABoVE campaign are shown as black dots (Hoy *et al*.^34^, and a boreal forest boundary derived from the ‘taiga’ portions of the World Wildlife Fund’s ecoregions map^57,58^ appears in dark gray. The ABoVE core (small-spaced) and extended domain (larger-spaced) dashed lines are also visible.

Arctic and boreal regions are experiencing rapid environmental changes, which have been observed by Northern communities^1–3^ and documented in scientific literature, including increased temperatures^4^, more frequent disturbances^5^, and vegetation shifts^6–9^. These changes have cascading impacts at local and global scales highlighting the importance of understanding Northern ecosystems^10^. The NASA Arctic-Boreal Vulnerability Experiment (ABoVE) aimed to expand observation and address knowledge gaps in these regions through a coordinated, decade-long campaign which combined field, remote sensing (airborne and space-borne), and modeling research^11^. A key component of this effort was the Land, Vegetation, and Ice Sensor (LVIS)^12^, a medium- to high-altitude (up to 10 km above ground) airborne laser altimeter providing critical data on vegetation structure and topography at spatial resolutions of 5–25 m. The LVIS airborne data record was collected during the 2017 and 2019 ABoVE Airborne Campaigns (AAC), covering extensive areas of Alaska and western Canada (Fig. 1). These data were acquired in similar seasons across both deployment years (29 June 2017 - 17 July 2017 and 12 July 2019 - 7 August 2019). Overall, the two years of LVIS deployments described in this collection represent the most biogeographically comprehensive airborne vegetation height and topography data record in North American boreal and Arctic regions, offering valuable opportunities for validating space-borne satellite products and supporting various ecological studies. This collection of data records provides a detailed understanding of the rapid environmental change occurring in these often remote boreal and tundra ecosystems.

LVIS, operated by Goddard Space Flight Center, uses a medium footprint (5–25 meters, depending on aircraft altitude) and waveform-digitizing technique to map vegetation height, structure, and topography with decimeter-level accuracy^12,13^. Its high-sensitivity waveforms can penetrate dense canopies^14^, making it ideal for shrubland and boreal forest studies^15^. LVIS data have been crucial in assessing carbon pools^16^, forest canopy-animal interactions^17^, aboveground biomass^18^, and forest growth patterns^19^ in various global locations. This instrument has been used in NASA airborne campaigns in the U.S.^12^, Costa Rica^14^, Greenland^20^, Antarctica^21^, Gabon^22^, and South Africa^23,24^.

The AAC was designed to capture the diverse geographical, ecological, and bioclimatic gradients present across North America’s boreal and Arctic regions^11^. It included foundational and investigator-led airborne measurements of imaging spectroscopy^25^, light detection and ranging (LiDAR)^12^, high-resolution optical camera imagery^26–28^, and Ka-, L- and P-band synthetic aperture radar (SAR)^29–31^. Flight locations were strategically planned based on the ABoVE Concise Experiment Plan^32^, ABoVE Implementation Plan^33^, and through consultation with individual research teams and cross-institution collaborations, incorporating input from over 6,700 field sites and remote sensing datasets^34^.

The LVIS data record collection serves multiple critical purposes within NASA’s Earth observation efforts. It provides scaling information linking field and satellite data, and acts as an airborne simulator and regional-scale validation^35^ for NASA’s Global Ecosystem Dynamics Investigation (GEDI)^36^ on the International Space Station (ISS). The data record collection’s geographic coverage importantly extends LiDAR observations northward of GEDI’s 51.6° N limit, while its strategic flight planning as part of the AAC collection included sampling along planned reference ground tracks of the NASA Ice, Cloud, and Land Elevation Satellite-2 (ICESat-2) instrument^37,38^. These coordinated observations support validation of ICESat-2 and other satellite-based laser altimeters that provide important data on many of Earth’s climate and physical variables^39,40^. The LVIS bare ground and canopy height data also provide critical absolute validation of other satellite-based remote sensing products, such as for the ArcticDEM, a 2 m digital surface model developed using optical stereo imagery^41^.

In boreal regions specifically, the ABoVE LVIS data record collection has supported multiple satellite-based research applications and validation efforts. The data record collection has been used to validate vegetation change models^15^ developed from ICESat-2 data, and was essential to validating a high spatial resolution global canopy height model trained on GEDI and Sentinel-2 observations^42^. LVIS data are also available for validation of an ICESat-2-derived aboveground biomass product for boreal forests^43^, and for recent research which modeled shifts in boreal vegetation^9^. Building on these validation applications, LVIS data have been integrated with ICESat-2 and satellite-based radar data to develop a forest canopy height model^44^. Looking toward future missions, the ABoVE LVIS data provide invaluable northern high-latitude observations for precursor studies of NASA’s potential Earth Dynamics Geodetic Explorer (EDGE) Earth System Explorer (ESE-1) mission^45^.

The data record collection described here encompasses various LiDAR and optical imagery data products, curated across NASA archives and accessible through NASA Earthdata^46^. This Data Descriptor paper details nine distinct data records from two deployment years of LVIS acquisition (2017 and 2019), including LiDAR surface and canopy data, optical camera imagery, and gridded vegetation structure data. The workflows used to produce these data records are also described.

The LVIS data record collection represents a unique and valuable resource for the scientific community. Its extensive coverage of Arctic and boreal North America, high accuracy, and integration with other NASA Earth observation efforts make it important for a wide range of applications. Its uses include mapping surface topography and creating high-resolution digital elevation models^47^. The LVIS data record collection is also well suited to studying vegetation dynamics^15,19,40,48–52^, assessing surface water hydrology^30^, and understanding ecosystem processes^53^ in these rapidly changing northern regions. The comprehensive nature of the ABoVE LVIS data record collection, combined with its strategic collection parameters, positions it as a crucial tool for researchers working to understand and model the complex environmental processes in Arctic and boreal ecosystems.

## Methods

Here we summarize the instruments, sampling strategy, and workflows used to create the LVIS data record collection for the AAC in 2017 (29 June 2017 - 17 July 2017) and 2019 (12 July 2019 - 7 August 2019). We first describe the LVIS instrument suite, including the imaging LiDAR and optical camera collection instruments. We then review the airborne data acquisition methodology used during the AAC. Lastly, we describe the LVIS data processing workflows for the different data product levels of LiDAR data and optical camera imagery.

### The LVIS instrument suite

#### LVIS Imaging LiDAR

The LVIS LiDAR instruments digitally record the shape of each outgoing and returning laser pulse^12^. The LVIS altimeters measure the time it takes for a pulse to reach a target object (such as the top of the canopy or ground) and return back to the sensor^24^. This time measurement is used to estimate the distance, or range, to the target. The range, along with information on the pointing and positioning of the laser, is used to determine the laser footprint on the ground relative to a reference ellipsoid (e.g., WGS-84)^54^. A signal digitizer, with a precise oscillator, is used by LVIS to measure the transmitted and reflected laser pulse energies over time (known as waveforms). GPS satellite data are used to determine laser positioning at the time of each laser shot. Laser pointing information is provided by an Inertial Measurement Unit (IMU) attached directly to the LVIS instrument.

The LVIS waveforms^13,14^ provide a three-dimensional (3D) representation of the surface structure, describing the relative positions of the various reflecting surfaces within each footprint, as well as their location relative to a reference ellipsoid (e.g., WGS-84)^54^. Waveforms can be simple or complex in nature, with a simple return waveform consisting of a single mode, meaning it produces one distinct peak in the recorded signal in the waveform, which occurs when the surface is relatively smooth within the laser footprint^24^. In contrast, a complex waveform contains more than one mode and is produced when the laser beam hits multilayered surfaces, such as vegetated land covers, or rocky terrain, returning multiple peaks. Additionally, different modes represent the various surfaces within the footprint, such as the canopy top and ground, and are distributed across their relative elevations within the footprint. These waveforms are used to develop the collection of LVIS data records, described below in the LVIS Data Processing Workflow and in the Data Records section.

Two versions of the LVIS imaging LiDAR instrument were flown in support of the AAC: LVIS-Facility and LVIS-Classic (Table 1). While both instruments generate nearly identical data products^55^, they differ in key operational parameters that affect their ground sampling characteristics. During the AAC, the LVIS-Facility instrument had a higher spatial resolution (nominal footprint diameter, 10 m) than LVIS-Classic (nominal footprint diameter, 25 m) (Table 1), making the LVIS-Facility instrument better suited for more detailed surface topography and vegetation structure analyses. In contrast, the LVIS-Classic instrument was specifically designed, and flown as part of the AAC, to provide LiDAR data directly comparable in spatial and spectral resolution to NASA’s GEDI^36^ LiDAR instrument aboard the ISS.

**Table 1:** Comparison table for the two versions of the LVIS LiDAR instrument (LVIS-Facility and LVIS-Classic) flown during the NASA ABoVE airborne campaign.

Characteristic	LVIS-Facility (LVISF) (2017 & 2019 flights)	LVIS-Classic (LVISC) (2019 flights only – GEDI comparison)
***Nominal Swath Width***	2017: 1.8 km (200 mrad) 2019: 2.5 km (200 mrad)	2.5 km (200 mrad)
***Nominal Footprint Diameter***	10 m (0.75 mrad)	25 m (2 mrad)
***Footprint Spacing***	7–10 m along and across track	20-25 m along and across track
***Laser Repetition Rate***	4,000 Hz	1,000 Hz
***Wavelength***	1064 nm wavelength, 5 ns laser	1064 nm wavelength, 9 ns laser

Both the LVIS-Facility and LVIS-Classic lasers operate at a wavelength of 1064 nm. However, the LVIS-Facility instrument operates with a faster repetition rate (4,000 Hz as compared to 1,000 Hz) and shorter pulse width than the LVIS-Classic instrument^56^, resulting in LVIS-Facility having a smaller ground sampling distance (and thus higher spatial resolution) when operated at the same altitude as the LVIS-Classic instrument. The LVIS-Facility uses a 5 ns laser pulse (full width at half maximum, FWHM) compared to LVIS-Classic, which uses a 9 ns laser pulse. The two LVIS instruments were co-mounted on the same platform and operated simultaneously during the AAC flights. From a nominal flight altitude of 10 km above ground level, LVIS-Facility projects a 7 m diameter footprint with a 2 km swath, while LVIS-Classic produces a 20 m diameter footprint. Both footprint and swath sizes can be adjusted through flight altitude changes to meet specific science requirements, which for the AAC included LVIS-Facility with a 10 m footprint, and LVIS-Classic with a 25 m footprint (Table 1).

During the 2017 LVIS deployment, only the LVIS-Facility LiDAR instrument was flown as part of the AAC, however during the 2019 LVIS deployment both versions of the LiDAR instrument were flown. In support of the ABoVE LVIS deployment in 2017, LVIS was flown on a Dynamic Aviation King Air B200T (N44U), and in 2019 LVIS was flown on a NASA Gulfstream-V (N95NA).

#### LVIS optical camera

**Fig. 2:**
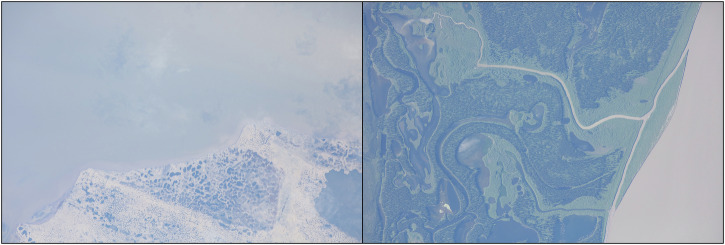
LVIS Level-1A Camera imagery. Left: polygonal land near the North Slope of Alaska (ABOLVIS1A_ABoVE2017_0709_R1802_075701.JPG), and Right: forested region in Canada adjacent to the Mackenzie River (ABOLVIS1A_ABoVE2017_0630_R1802_087103.JPG).

The LVIS sensor suite also includes high-resolution digital cameras to provide context for the LiDAR data. The LVIS cameras flown during the ABoVE LVIS deployments in 2017 and 2019 (Canon EOS 5DS R) used 50 Megapixel sensors configured with two different focal length telephoto lenses, were mounted adjacent to the LVIS LiDAR instrument, faced downward during each flight, and included along-track image overlap. In 2017, a single camera (LVISCAM1) collected optical imagery, while in 2019, two cameras were used: LVISCAM1 mounted next to the LVIS-Facility instrument, and LVISCAM2 mounted next to the LVIS-Classic instrument. Sample imagery from the LVIS camera used in 2017 is provided in Fig. 2, while specifics of the camera system setup and imagery can be found in Table 2. In general, while the camera model was the same across both years, there were slight differences between the lenses, image resolution, nominal resolutions, and nominal overlaps between the cameras.

**Table 2:** LVIS Camera imagery specifications and information.

Characteristic	2017 Deployment	2019 Deployment	
*Camera Model*	Canon EOS 5DS R	Canon EOS 5DS R	
*Camera Name*	LVIS-Camera 1	LVIS-Camera 1	LVIS-Camera 2
***Lens Model***	Carl Zeiss Makro-Planar T* 100 mm f/2 ZE	Carl Zeiss Makro-Planar T* 100 mm f/2 ZE	Carl Zeiss Planar T* 85 mm f/1.4 ZE
***Image Resolution***	50.3 Megapixels 8688 px × 5792 px	50.3 Megapixels 8688 px × 5792 px	50.3 Megapixels 5792 px × 8688 px
***Nominal Resolution***	3.1 km × 2.0 km (0.35 m/px)	4.2 km × 2.8 km (0.5 m/px)	3.3 km × 4.9 km (0.6 m/px)
***Nominal Overlap***	75%	67%	80%
***Camera Product Designation***	LVISCAM1	LVISCAM1 (mounted next to LVIS-Facility)	LVISCAM2 (mounted next to LVIS-Classic)

### ABoVE LVIS flights data acquisition

ABoVE LVIS airborne deployments occurred in the summers of 2017 (29 June 2017 - 17 July 2017) and 2019 (12 July 2019 - 7 August 2019). Each deployment captured the region’s dynamic changes across geographical locations and the interannual variability of the landscapes and ecosystems under study^11,57,58^. These deployments overlapped with other ABoVE airborne instrument flight campaigns^11,25,29,30^. Over 91,000 km^2^ were imaged by LVIS throughout the two years (Table 3). Flight paths in 2019 were largely designed to cover areas not acquired during the 2017 flights (Fig. 1), prioritizing expanded coverage over repeat measurements. Despite this focus on one-time sampling, some areas were observed during both years, resulting in 4,234 km^2^ (9% of the total area imaged) with overlapping coverage. In total, data were collected on 17 flights in 2017 and 15 flights in 2019 (Tables 4, 5), for a total of 32 flights across both deployments. Notable differences between the two years included the sensor platform, altitude flown, and total area imaged (Table 3).

**Table 3:** Comparison between the 2017 and 2019 LVIS airborne collections.

Characteristic	2017 Deployment	2019 Deployment
***Dates of Deployment***	29 Jun 2017–17 Jul 2017	12 Jul 2019–07 Aug 2019
***Altitude of Sensor***	~8,000 m	~13,000 m
***Platform***	Dynamic Aviation King Air B200T	NASA Gulfstream V
***LVIS Sensor Flown***	LVIS-Facility	LVIS-Facility, LVIS-Classic
***Number of Flights***	17	15
***Total Length of Flight Lines***	22,913 km	65,282 km
***Total Area imaged***	16,900 km²	74,494 km²

The total length and area of flight lines are derived from the ABoVE LVIS L3 footprints, from Montesano *et al*.^76^.

**Table 4:** The 17 LVIS flight dates and locations from the 2017 deployment.

Modified Julian Date (MJD)	Date	Region / Remarks
57933	29 Jun 2017	Saskatoon, Saskatchewan to Yellowknife, Northwest Territories, Canada
57933	29 Jun 2017	Great Slave Lake, Northwest Territories, Canada
57934	30 Jun 2017	Yellowknife to Inuvik, Northwest Territories, Canada
57934	30 Jun 2017	Inuvik to Yellowknife, Northwest Territories, Canada
57935	1 Jul 2017	Daring Lake, Northwest Territories, Canada
57936	2 Jul 2017	West and Southwest Great Slave Lake, Northwest Territories, Canada
57937	3 Jul 2017	Yellowknife, Northwest Territories to Whitehorse, Yukon, Canada
57937	3 Jul 2017	Whitehorse, Yukon, Canada to Fairbanks, Alaska
57940	6 Jul 2017	Kluane Lake, Yukon, Canada
57941	7 Jul 2017	Healy, Alaska
57943	9 Jul 2017	Fairbanks to Barrow, Alaska
57948	14 Jul 2017	Fairbanks to Deadhorse, Alaska via Toolik Lake Long Term Ecological Research (Toolik LTER) Station
57948	14 Jul 2017	Deadhorse to Fairbanks, Alaska via Fort Yukon
57949	15 Jul 2017	Fort Yukon, Alaska
57950	16 Jul 2017	Fairbanks to Ketchikan, Alaska
57950	16 Jul 2017	Ketchikan, Alaska to Glasgow, Montana
57951	17 Jul 2017	Boreal Ecosystem Research and Monitoring Sites (BERMS) flight, Saskatchewan, Canada

The Region/Remarks field provides details on the targets of interest the flight imaged, based on contributions from the ABoVE Science Team.

**Table 5:** The 15 LVIS flight dates and locations from the 2019 deployment.

Modified Julian Date (MJD)	Date	Region / Remarks
58676	12 Jul 2019	Houston, Texas to Yellowknife, Northwest Territories, Canada; GEDI reference ground tracks
58677	13 Jul 2019	BERMS, Saskatchewan, Canada; Peace Athabascan Delta (PAD), Alberta, Canada; 2010 LiDAR^74^ repeat
58679	15 Jul 2019	Mackenzie River, Norman Wells, Scotty Creek Research Station (SCRS), and Great Slave Lake, Northwest Territories, Canada; ICESat-2 tracks
58680	16 Jul 2019	Daring Lake and Great Slave Lake, Northwest Territories, Canada; PAD, Alberta, Canada; 2010 LiDAR repeat
58682	18 Jul 2019	Yellowknife, Northwest Territories, Canada to Fairbanks, Alaska; Kakisa Lake and SCRS, Northwest Territories, Canada; Delta Junction, Alaska; ICESat-2 tracks; 2010 LiDAR repeat
58686	22 Jul 2019	Healy, Bonanza Creek Long Term Ecological Research Station (BNZ LTER), Yukon Flats, and Delta Junction, Alaska
58687	23 Jul 2019	Southwest Alaska; Central Interior Alaska
58689	25 Jul 2019	Northern Alaska including Prudhoe Bay, Alaska; Delta Junction, Alaska; 2019 Shovel Creek Fire Event near Fairbanks, Alaska (Shovel Creek Fire); Old Crow, Yukon; Mackenzie River and Inuvik, Northwest Territories, Canada
58691	27 Jul 2019	Seward Peninsula, Shovel Creek Fire, and Delta Junction, Alaska
58692	28 Jul 2019	Seward Peninsula, Yukon-Kuskokwim Delta (YK Delta), and Shovel Creek Fire, Alaska
58693	29 Jul 2019	Western Alaska, Utqiagvik, Nuisqut, and Shovel Creek Fire, Alaska; ICESat-2 tracks
58695	31 Jul 2019	Fort Watson, Kluane Lake, and Whitehorse,Yukon; ICESat-2 tracks
58696	01 Aug 2019	ICESat-2 tracks; 2010 LiDAR repeat; Shovel Creek Fire
58702	06 Aug 2019	Fairbanks, Alaska to Salt Lake City, Utah; Wind River Experimental Forest, Washington; ICESat-2 tracks; GEDI reference ground tracks
58702	07 Aug 2019	Salt Lake City, Utah to Houston, Texas; GEDI reference ground tracks

The Region/Remarks field provides details on the targets of interest the flight imaged, based on contributions from the ABoVE Science Team.

ABoVE science team members strategically selected LVIS acquisition locations to maximize collection with complementary airborne, ground-based, and satellite measurements^11,34^ (see Tables 4, 5 for regions of interest and remarks for each flight). This coordinated approach enables multiple types of sensor comparisons and integrated analyses across the ABoVE domain. For example, as LVIS LiDAR data provide detailed 3D bare ground and vegetation structure information, these data can be compared with airborne and satellite-based SAR measurements. Comparisons with SAR have proven valuable for evaluating differences in forest height and aboveground biomass measurements—an established approach in boreal forests^59,60^ and other ecosystems^61,62^. Beyond biomass applications, LiDAR and SAR comparisons also support investigations of environmental disturbances, including permafrost thaw, subsidence, and fire disturbance^63^.

Building on traditional SAR capabilities, tomographic SAR (tomoSAR) uses multiple SAR images taken from different positions or times to extend traditional 2D SAR capabilities into 3D surface structure mapping. This advancement allows for height-based change detection products that can be directly compared with the LVIS 3D surface and vegetation data^22,29^. For the ABoVE campaign, comparisons between LVIS and SAR or tomoSAR data are ongoing, including in Delta Junction, Alaska^64,65^, and at the Boreal Ecosystem Research and Monitoring Sites (BERMS) near Prince Albert, Saskatchewan, Canada^29,66–68^. The LVIS data at BERMS have also supported intercomparisons with the German Aerospace Center (DLR) F-SAR instrument^66^. Furthermore, when combined with airborne interferometric Ka-band SAR (Air Surface Water and Ocean Topography, or AirSWOT), the detailed LVIS ground surface and height data enable assessment of water surface elevation differences^30,31,69^.

In addition to SAR, the LVIS acquisitions were also designed to co-align with another foundational instrument of the AAC, the Airborne Visible / Infrared Imaging Spectrometer (AVIRIS-NG and AVIRIS-3) flights^25^. These coordinated observations advance mapping and monitoring of plant diversity across the ABoVE domain, and ultimately facilitate the differentiation of structurally different plant species or plant functional types (PFTs). Integration of LVIS and AVIRIS data records is also underway to characterize insect-disturbed forests within the ABoVE domain^70^.

The 3D structure data from LVIS have other potential and ongoing applications across the ABoVE domain. For example, LVIS data are being used to understand snow-vegetation interactions, a critical process undergoing significant changes with increasing temperatures and shrubification^71,72^. Others are using the LVIS data record collection to assess plant productivity variation across the forested extents studied in ABoVE^19^, to calibrate Landsat-based estimates of boreal tree canopy cover^51^, and to validate drone-based estimates of canopy height^52^. LVIS imaged a recently burned fire event near Fairbanks, Alaska in July 2019 (Shovel Creek Fire)^73^, offering a unique opportunity to collect post-fire data for analysis (Table 5). Additionally, the 2019 LVIS data collection included North-South transects along planned ICESat-2 reference ground tracks (Fig. 1) and repeated 2010 airborne LiDAR tracks collected by a different sensor^74^ to enable cross-platform comparisons and expand geographic coverage for science applications.

**Fig. 3:**
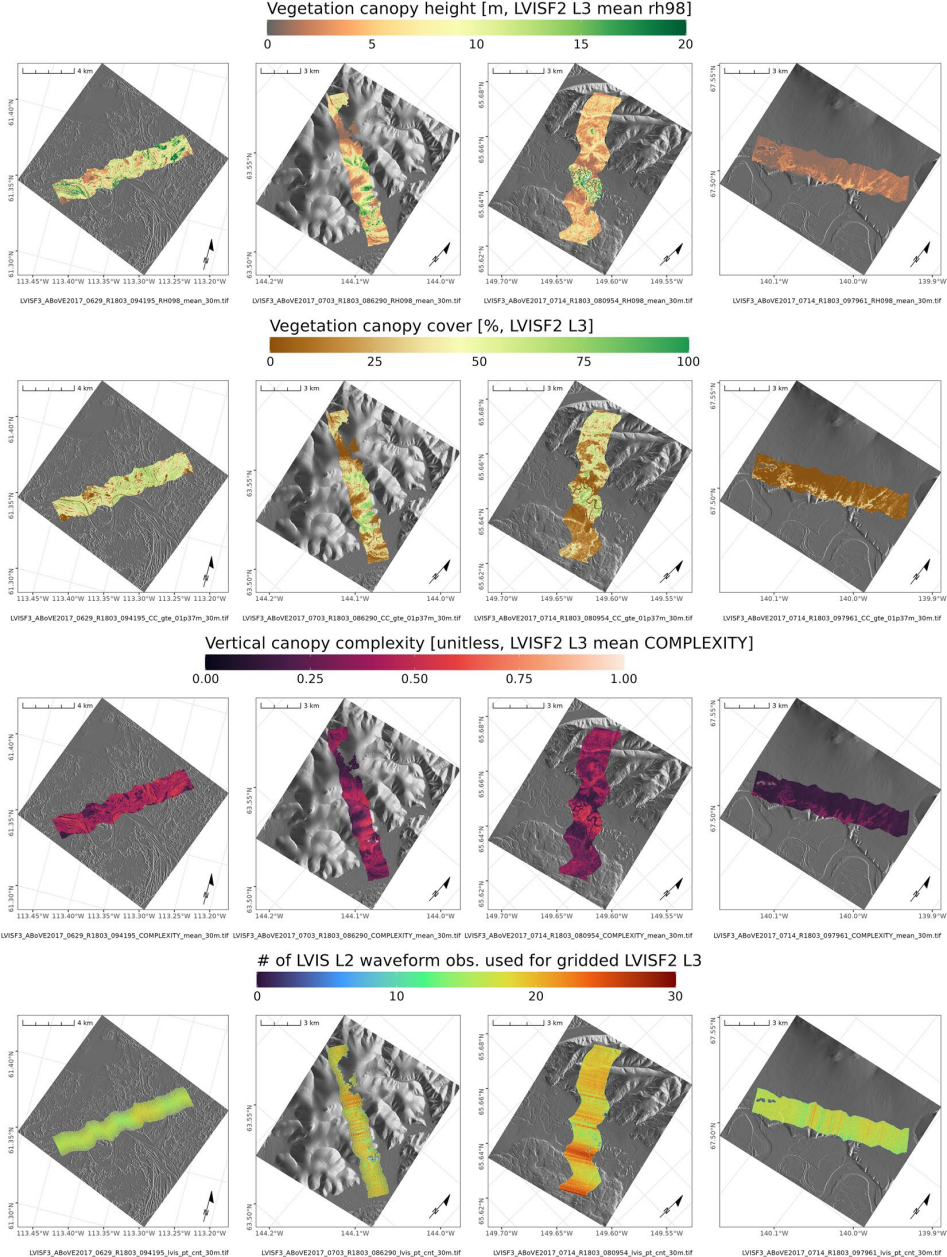
LVIS vegetation canopy height, vegetation canopy cover, vertical canopy complexity, and observations for selected areas within the ABoVE domain, developed from the ABoVE LVIS Level-3 (L3)^76^ data record and based on the ABLVIS2^81^ and LVISF2^24^ data records.

The ABoVE LVIS acquisitions resulted in a collection of LiDAR-derived data records including geolocated laser waveform data for each laser footprint, canopy top and ground elevations, relative canopy height, and gridded LiDAR-derived data (Fig. 3). Flights from both deployments (2017 and 2019) also include high-resolution camera imagery (see Fig. 2 and Table 2).

### LVIS Data processing workflow

**Fig. 4:**
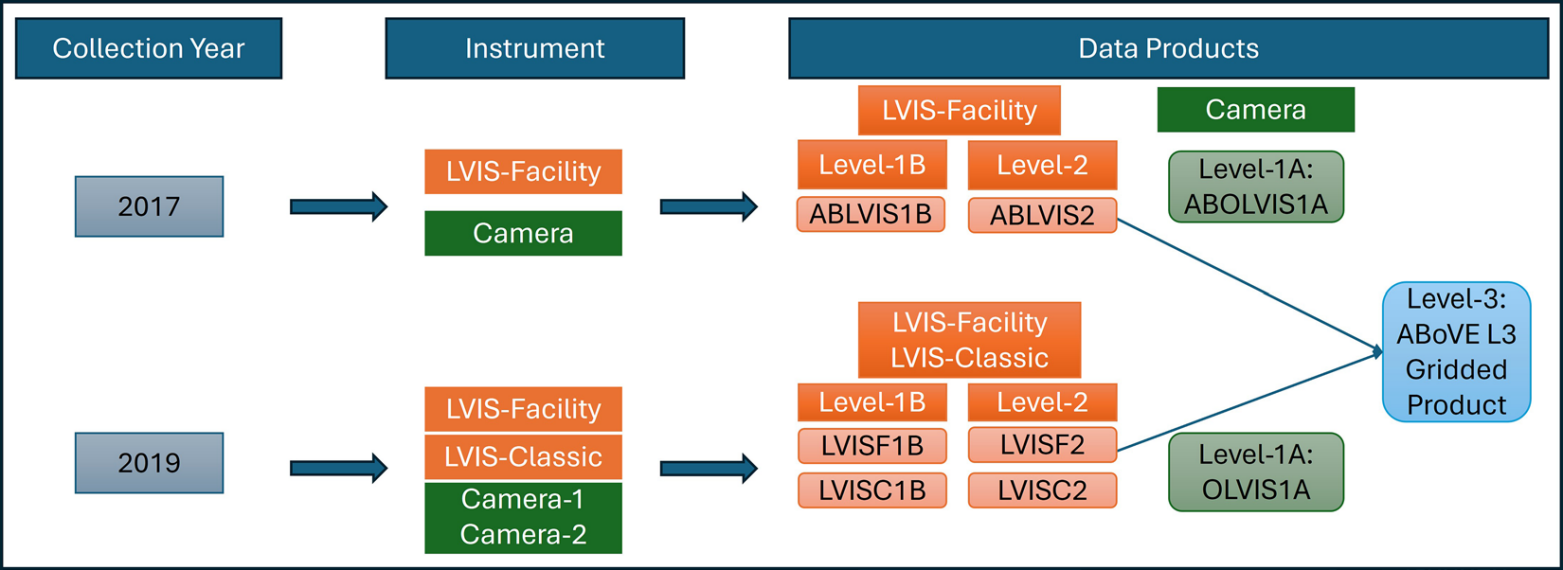
An overview of the data records available for the ABoVE LVIS collection from 2017 and 2019. See Table 6 and the Data Records section for detailed descriptions of each data record.

**Fig. 5:**
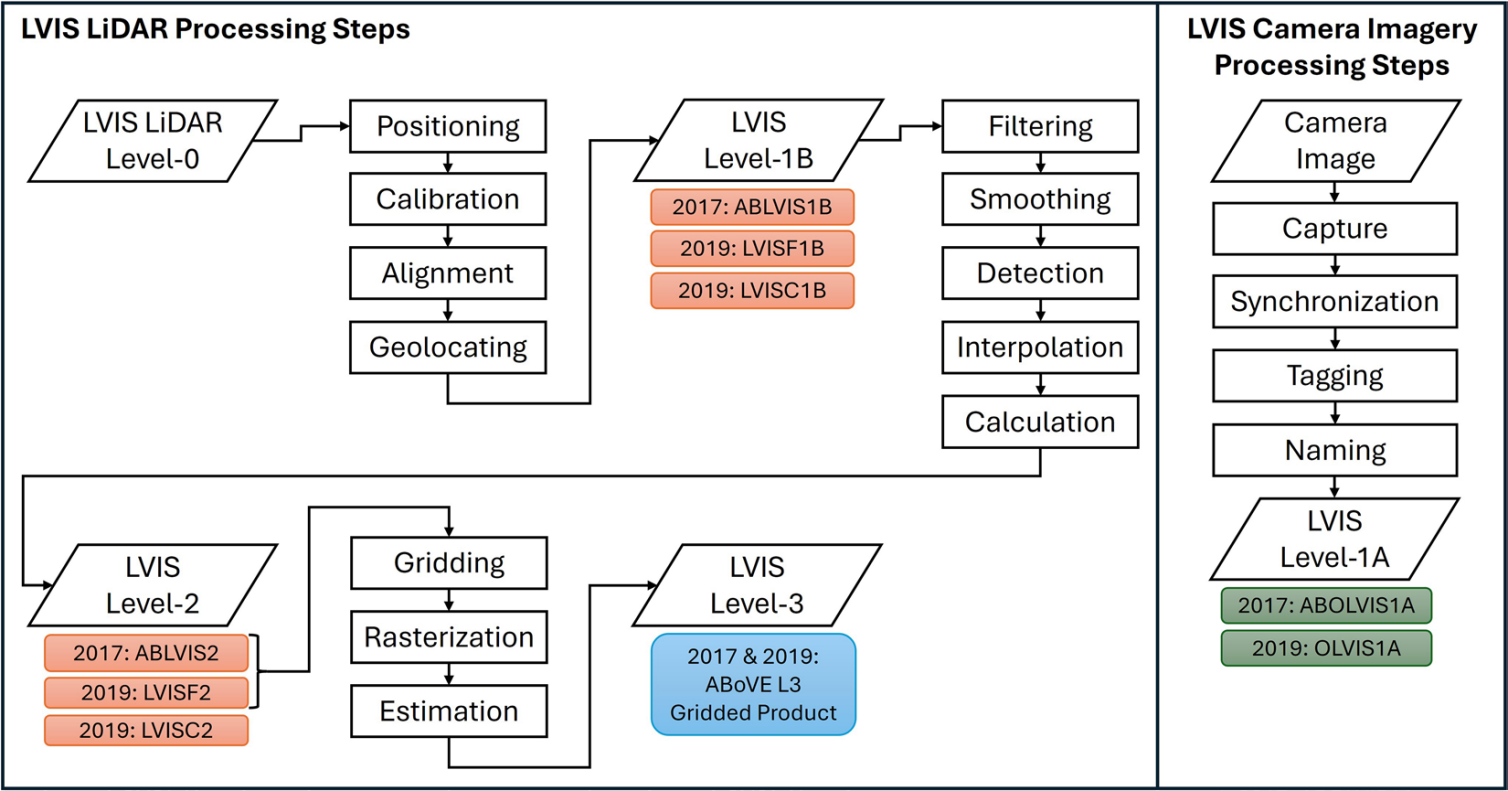
Processing workflow for the ABoVE LVIS LiDAR and camera imagery data records from 2017 and 2019, showing data records produced at each processing level. See Table 6 and the Methodology section for additional details.

The collection of LVIS data records generated for ABoVE is provided in Table 6 and Fig. 4, and the data processing workflow is shown in Fig. 5. Table 6 includes an abbreviated Data Record ID, full data record title, temporal coverage for ABoVE data, sensor used, parameters collected, and data format. The data records vary by data processing level^75^ and include data records in Level-1A, Level-1B, Level-2, and Level-3, although not all processing levels are provided for each type of data record in the collection (i.e. Level-1A imagery is only available for camera imagery). In general, NASA has a structure for data record processing levels where Level-1A NASA data records contain reconstructed, unprocessed instrument data with ancillary information, Level-1B data include Level-1A data that have been processed to instrument units, Level-2 data include derived geophysical variables at the same resolution and location as the Level-1 source data, and Level-3 data include variables mapped on uniform space-time grid scales^75^.

**Table 6:** LVIS LiDAR and camera imagery data records available at two NASA archive centers (NSIDC DAAC and ORNL DAAC), although all can be found through NASA Earthdata^46^.

Data Record ID	Title/URL	Temporal Coverage for ABoVE Data	Sensor(s)	Parameter(s)	Data Format
***LiDAR Data Records at NSIDC DAAC***					
ABLVIS1B^78^	ABoVE LVIS L1B Geolocated Return Energy Waveforms, Version 1 10.5067/UMRAWS57QAFU	2017-06-29 to 2017-07-17	Altimeters, Lasers, LVIS	Sensor Counts	HDF5
ABLVIS2^81^	ABoVE LVIS L2 Geolocated Surface Elevation Product, Version 1 10.5067/IA5WAX7K3YGY	2017-06-29 to 2017-07-17	Altimeters, Lasers, LVIS	Terrain Elevation	ASCII
LVISC1B^79^	LVIS Classic L1B Geolocated Return Energy Waveforms, Version 1 10.5067/O8UCOA2D6ZE3	2019-07-12 to 2019-08-07	LVIS	Sensor Counts	HDF5
LVISC2^56^	LVIS Classic L2 Geolocated Surface Elevation and Canopy Height Product, Version 1 10.5067/W569D47GCOUX	2019-07-12 to 2019-08-07	LVIS	Terrain Elevation, Canopy Height	ASCII
LVISF1B^80^	LVIS Facility L1B Geolocated Return Energy Waveforms, Version 1 10.5067/XQJ8PN8FTIDG	2019-07-12 to 2019-08-07	LVIS	Sensor Counts	HDF5
LVISF2^24^	LVIS Facility L2 Geolocated Surface Elevation and Canopy Height Product, Version 1 10.5067/VP7J20HJQISD	2019-07-12 to 2019-08-07	LVIS	Terrain Elevation, Canopy Height	ASCII
***LiDAR Data Records at ORNL DAAC***					
ABoVE LVIS L3^76^	ABoVE: LVIS L3 Gridded Vegetation Structure across North America 10.3334/ORNLDAAC/1923	2017-06-29 to 2017-07-17 and 2019-07-12 to 2019-08-07	LVIS	Gridded LVIS Level-2 Data, Canopy Cover derived from Level-2 Data	GeoTIFF, Geopackage
***Camera Imagery Data Records at NSIDC DAAC***					
ABOLVIS1A^27^	ABoVE LVIS L1A Geotagged Images, Version 1 10.5067/4O5WY1ORYWK2	2017-06-29 to 2017-07-17	LVIS- Camera	Visible Imagery	JPEG
OLVIS1A^28^	LVIS L1A Geotagged Images, Version 1 10.5067/NE5KKKBAQG44	2019-07-12 to 2019-08-07	LVIS- Camera	Visible Imagery	JPEG

For the LVIS collection of data records, data processing levels have been tailored to the type of record collected. Only the LVIS camera imagery files (in JPEG format) are delivered at Level-1A, where the header file has been modified to include camera pointing/positioning information. Level-1B and Level-2 data are available for the LVIS waveform LiDAR data records. The Level-1B laser data are geolocated laser return waveforms, which include waveforms with latitude, longitude, and elevation (relative to the WGS-84 datum) of both the highest and lowest bins provided in the data. The Level-1B data are then processed to provide the Level-2 bare ground elevation and vegetation canopy heights from the geolocated laser return waveforms.

Level-1 (LiDAR data and camera imagery) and Level-2 (LiDAR data) data records are provided through the National Snow and Ice Data Center Distributed Active Archive Center (NSIDC DAAC, https://nsidc.org/data/lvis/data). Montesano *et al*. created an LVIS LiDAR Level-3 data record^76^ that reprojects the Level-2 vegetation structure data into the common grid and projection used by the ABoVE campaign^77^; this data record is available in GeoTIFF format through the Oak Ridge National Lab Distributed Active Archive Center (ORNL DAAC, 10.3334/ORNLDAAC/1923). While different NASA archives curated and published these LVIS data records, all LVIS data records can be publicly accessed through NASA Earthdata^46^. See the Data Records section for additional details on each data record in the LVIS collection.

We provide additional details about the data processing workflow for each of the LVIS data records below and in Figs. 4, 5. The data record archive at the NSIDC DAAC or ORNL DAAC includes a detailed user guide with information on data acquisition and processing workflows for each of these data records.

#### Level-1B LiDAR data processing: 2017 Level-1B (ABLVIS1B), 2019 Level-1B Classic (LVISC1B), and 2019 Level-1B Facility (LVISF1B)

The three Level-1B LiDAR data records described here include^78–80^ the 2017 ABoVE LVIS Level-1B Geolocated Return Energy Waveforms (ABLVIS1B)^78^, 2019 LVIS Classic Level-1B Geolocated Return Energy Waveforms (LVISC1B)^79^, and 2019 LVIS Facility Level-1B Geolocated Return Energy Waveforms (LVISF1B)^80^. The Level-1B data processing methodology is consistent between these three data records across 2017 and 2019, with data records generated from raw (Level-0) instrument data using the following processing steps (see processing workflow presented in Fig. 5):

1. **Positioning:** The GPS and IMU data are post-processed to generate the airplane positioning and pointing information. These data streams can be processed in multiple ways, such as differential kinematic or precise point positioning GPS that are loosely or tightly coupled with the IMU data. The resulting positioning and attitude data are then merged with the laser data to produce the latitude, longitude, altitude, roll, pitch, and heading of the airplane for each laser shot.
2. **Calibration:** The laser range measurement is calculated based on the travel time of the laser pulse from the laser reference frame origin to the surface and back to the instrument receiver. The range is adjusted for delays associated with internal system responses (e.g., cabling lengths), which are determined by calibration experiments that are typically performed in the lab before the deployment. An atmospheric correction is also applied to each laser measurement. This adjustment is necessary because temperature and pressure affect the speed of light through the atmosphere. The correction is computed using a model and data (standard temperature and pressure) extrapolated from the nearest meteorological station. Additional checks to a target surface of known elevation may be performed during a flight.
3. **Alignment:** Measurement model parameters to align the various reference frames are determined. These include angular offsets between the IMU and laser reference frames, translation to relocate the GPS measurements at the laser reference frame origin, and timing biases between the IMU and the laser. Estimates for angular measurement model parameters can be determined by flying the airplane through controlled roll and pitch maneuvers over a known, preferably flat, surface. The offset between the GPS antenna and the laser reference frame origin is found by performing a static GPS survey between several system components inside and outside the grounded airplane.
4. **Geolocating:** The laser position and pointing vectors as well as the measurement parameters are input to the measurement model to transform the laser range from a local reference system within the airplane to a global reference frame and ellipsoid, thus creating a geolocated data record. For the Level-1B data records, the ranges between 2 reference points are geolocated corresponding to the highest and lowest records of the laser return waveform.

For more details see Hofton *et al*.^54^.

#### Level-2 LiDAR data processing: 2017 Level-2 (ABLVIS2), 2019 Level-2 Classic (LVISC2), and 2019 Level-2 Facility (LVISF2)

The three Level-2 LiDAR data records described here include^24,56,81^ the 2017 ABoVE LVIS Level-2 Geolocated Surface Elevation Product (ABLVIS2)^81^, 2019 LVIS Classic Level-2 Geolocated Surface Elevation and Canopy Height Product (LVISC2)^56^, and 2019 LVIS Facility Level-2 Geolocated Surface Elevation and Canopy Height Product (LVISF2)^24^. The Level-2 data processing methodology is consistent between 2017 and 2019, with each derived from their corresponding Level-1B geolocated return energy waveform data records: 2017 Level-2 (ABLVIS2)^81^ from ABLVIS1B records, and 2019 Level-2 Classic (LVISC2)^56^ and Facility (LVISF2)^24^ from LVISC1B and LVISF1B records, respectively. The following processing steps are performed by the data provider to produce the Level-2 data (see processing workflow presented in Fig. 5):

1. **Filtering:** Establish noise threshold settings to be applied to each Level-1B waveform based on background noise threshold levels, then establish the area within each laser return waveform to search for surface signals.
2. **Smoothing:** De-noise (smooth) the laser return waveform by convolution with a Gaussian function. This step maximizes the signal to noise ratio.
3. **Detection:** Determine surface timing/ranging points (e.g., lowest, highest detected signals, center of each reflected mode) and energy metric locations relative to the start of each laser return waveform^13,20^.
4. **Interpolation:** Linearly interpolate the geolocation information provided in the Level-1B data to the surface timing/ranging points determined in step 3 to generate the geolocation of the Level-2 data records (footprint positioning is at the 1–2 m level)^82^. Relative height data records are then computed relative to the elevation of the lowest detected mode.
5. **Calculation:** Calculate additional surface and range-related parameters such as waveform complexity^83^, energy, sensitivity, laser beam incident angle and azimuth^82^.

For more details on the waveform analysis algorithms and derivation of footprint level data, and see Hofton and Blair (2019)^82^.

#### Level-3 data processing for gridded 2017 and 2019 data record (ABoVE LVIS L3)

The gridded Level-3 LVIS data record (ABoVE: LVIS L3 Gridded Vegetation Structure across North America^76^, ABoVE LVIS L3; Fig. 1, Table 6) uses the 2017 and 2019 LVIS-Facility Level-2 data records (see the data records (Fig. 4) and processing workflow (Fig. 5) for additional details). LVIS-Classic instrument data were not used to generate this data record. The Level-3 data record includes canopy cover estimates (CC), vertical canopy complexity metrics (COMPLEXITY), canopy height metrics (RH), mean, minimum, and maximum terrain elevation (ZG), and the number of footprints per pixel (pt_cnt) (Fig. 3 and Table 7). For additional details, refer to Montesano *et al*.^19^.

**Table 7:** ABoVE LVIS Level-3 data record variable names (GRIDNAME) and descriptions for the GeoTIFF files.

Variable (GRIDNAME)	Rasterization Function (STAT)	Data Type	Description
lvis_pt_cnt	Count	Byte	The count of footprints whose centroids intersected the grid cell (the per-pixel count of the number of LVIS footprints available to produce a pixel’s estimate). There is one file per flightline. There are a total of 3,579 files.
ZG	Minimum, Mean, Maximum	Float32	The ground surface elevation in meters. There are three files per flightline (mean, maximum, and minimum). The ‘mean’ was calculated using the mean of the ZG values per pixel, the ‘max’ using the maximum of the ZG values per pixel, and the ‘min’ using the minimum of the ZG values per pixel. There are 10,737 ZG files.
COMPLEXITY	Mean	Float32	The number of surfaces detected in the waveform, also described as vertical canopy complexity. There is one file per flightline for a total of 3,441 files.
RH < metric_value > Metric_values: 010,015,020,025,030,035,040,045, 050,055,060,065,070,075,080,085,090,095,096,097,098,099,100	Mean	Float32	Relative height percentiles of canopy surfaces above the ground, in meters. There are 23 files per flightline for a total of 82,317 files.
CC_gte_ < height_threshold > Height_thresholds: 00p20, 00p30, 00p50, 00p75, 01p00; 01p37, 01p50, 02p00, 03p00, 04p00, 05p00, 06p00, 07p00, 08p00, 09p00, 10p00, 12p00, 15p00	Mean	Uint16	The estimated canopy cover at a height greater than or equal to the specified height threshold in meters (e.g., 01p37 = 1.37 m). Values are scaled by 10000; for example 5000 represents a canopy cover of 0.5 or 50%. There are 18 files per flightline for a total of 64,404 files.

#### Gridding individual footprint estimates of vegetation structure

**Gridding:** The gridded footprint estimates of vegetation structure summarize the vertically continuous LiDAR waveform. They include heights of canopy components at statistical percentiles recorded along the vertical distribution of LiDAR energy returned to the sensor, and are referenced as heights above a detected ‘ground’ mode. Footprint gridding was handled according to ‘flightline’, an organizational spatial grouping of footprints associated with along-track subsets of airborne data collection tracks.

For each flightline, the ground latitude and longitude fields were used to assign spatial coordinates to the center of each footprint. The “raster” package in R (version 3.6.1) was used to initialize an empty raster grid to 30 m resolution in the Canada Albers Equal Area Conic projection (EPSG:102001). This base grid was aligned to the ABoVE 30 m standard reference grid^77^. A set of vertical and horizontal vegetation structure and surface topography attributes and estimates were calculated for each grid cell from all associated footprints. A grid cell’s associated footprints were those with centroids located within the 30-m grid cell.

**Rasterization:** Each flightline’s footprints were summarized to this raster grid with a 30 m resolution using a rasterization function (‘mean’), and each flightline’s grid was exported in GeoTIFF format. The number of GeoTIFF files for each flightline is associated with the number of gridded attributes calculated from the Level-2 data. These include relative canopy height (RH) and canopy cover (CC) metrics, a vertical canopy complexity estimate (COMPLEXITY)^83^, a bare ground elevation estimate (ZG), and a count of all the contributing footprints (‘count’) to each grid cell’s calculation. A ‘min’ and ‘max’ ZG gridded estimate for each cell was also included. Caution should be applied to interpreting the data in cases when the range of ZG values is of a similar magnitude to the RH values, or the CC threshold. Table 7 describes each variable.

#### Estimating vegetation canopy cover

**Estimation:** The Level-3 gridded data also include a suite of vegetation canopy cover estimates calculated for each footprint before gridding but which are not available in the Level-2 data. These estimates characterize the horizontal component of vegetation structure. For each footprint, estimates of canopy cover (*CC_gte_ < height_threshold > *) were calculated as 1.0 minus the lowest relative height metric (a quantile value) whose height value exceeded a height threshold. The result was an estimate of the proportion of the returned energy derived from above a specified height, all of which was assumed to be the vegetation canopy. Some components of this metric include signals from the ground return, especially for low height bins (e.g. < 1.37 m on flat terrain). Estimates of canopy cover were made for a suite of height thresholds from 0.2–15.0 m.

#### Level-1A Camera Imagery: 2017 (ABOLVIS1A) and 2019 (OLVIS1A) processing

The two Level-1A camera imagery data records described^27,28^ here include the 2017 ABoVE LVIS Level-1A Geotagged Images (ABOLVIS1A)^27^ and 2019 LVIS Level-1A Geotagged Images (OLVIS1A)^28^. See the processing workflow presented in Fig. 5, and sample imagery in Fig. 2, for more details.

**Capture**: Images were taken with one or two downward-facing (nadir) Canon EOS cameras (see Table 2 for more information) and stored via Ethernet on a supporting computer running the Canon EOS camera utility software to monitor and control image exposure.

**Synchronization**: Frame capture was controlled using an external intervalometer, which provided a Transistor-Transistor-Logic (TTL) pulse to the Applanix Position and Orientation system (navigation system), enabling precise timing, positioning, and attitude data to be added to each image during post-processing. The intervalometer rate varied depending on the altitude and ground speed of the airplane and desired overlap between images, with images acquired at 5-second intervals in this case.

**Tagging**: Each image was tagged with acquisition metadata including GPS time stamp, GPS date stamp, latitude, longitude, altitude, roll, pitch, and yaw (GPS image direction).

**Naming**: The image filename was amended to include the acquisition time in number of seconds since UTC/GPS midnight of the day on which data collection started.

## Data Records

All data records described within this descriptor have been published and archived at a NASA archive center. The NSIDC DAAC (https://nsidc.org/data/lvis/data) holds the permanent archive of LVIS LiDAR and camera data records across all NASA campaigns including the ABoVE LVIS Level-1B LiDAR data (ABLVIS1B^78^, 10.5067/UMRAWS57QAFU; LVISC1B^79^, 10.5067/O8UCOA2D6ZE3; LVISF1B^80^, 10.5067/XQJ8PN8FTIDG) and Level-2 LiDAR data (ABLVIS2^81^, 10.5067/IA5WAX7K3YGY; LVISC2^56^, 10.5067/W569D47GCOUX; LVISF2^24^, 10.5067/VP7J20HJQISD) and Level-1A camera imagery (ABOLVIS1A^27^, 10.5067/4O5WY1ORYWK2; OLVIS1A^28^, 10.5067/NE5KKKBAQG44). The ABoVE LVIS Level-3 gridded data record of vegetation structure, derived from the Level-2 LiDAR data records, is available from the ORNL DAAC (ABoVE LVIS L3^76^, 10.3334/ORNLDAAC/1923), which holds a majority of the field and remote sensing data records developed for ABoVE (find the full collection of ABoVE data records held at the ORNL DAAC here: https://www.earthdata.nasa.gov/data/projects/above). The LVIS LiDAR Level-1B data records are provided in HDF5 format, the Level-2 LiDAR data records in ASCII format, the ABoVE LVIS gridded vegetation structure data in GeoTIFF or Geopackage format, and the Level-1A camera imagery data records in JPEG format. For more details on the available data records, specific names of each data record, and data record formats, see Table 6 and Fig. 4. Additionally, LVIS flight and camera trajectories from the ABoVE campaign are available from the LVIS instrument website hosted by Goddard Space Flight Center^84,85^. All NSIDC DAAC and ORNL DAAC LVIS data records are being migrated to NASA Earthdata^46^ (https://www.earthdata.nasa.gov/), as NASA moves their complete catalog of Earth Data to an accessible open-access archive.

### ABoVE LVIS Level-1B data from 2017 (ABLVIS1B) and 2019 (LVISC1B and LVISF1B)

The ABoVE LVIS Level-1B data files^78–80^, in HDF5 format, contain the geolocated laser return waveform vector data for each laser footprint. These Level-1B data records enable end users to derive elevation and other data records as needed as the data record maintains full accuracy of the system and allows end-user interpretation and precise re-analysis.

### ABoVE LVIS Level-2 data from 2017 (ABLVIS2) and 2019 (LVISC2 and LVISF2)

The ABoVE LVIS Level-2 data files^24,56,81^, in ASCII format, contain surface elevation, heights and structure data records derived from the Level-1B data using standard LVIS algorithms as described in the Usage Notes and Code Availability sections (including canopy top and ground elevations, and relative heights, see Hofton and Blair (2019)^82^). This Level-2 data record provides the latitude, longitude, and elevation of the center of the lowest mode in waveform (mean ground elevation), the latitude, longitude, and elevation of the highest detected surface, the latitude, longitude, and elevation of the center of the highest detected mode in the waveform and vertical structure metrics (RH10 through RH100 at 5% intervals) based on energy quartiles, with the energy in lowest mode and waveform complexity^83^ (Fig. 3).

### ABoVE LVIS Level-3 gridded 30 m data (ABoVE LVIS L3)

The LVIS LiDAR Level-3 data record^76^, in GeoTIFF or Geopackage format, is a gridded data record of vegetation structure (e.g., canopy height, cover, and complexity) and topography characteristics available from the ORNL DAAC. These data are discoverable and accessible via download or on-the-fly cloud access at ORNL’s Amazon Web Service’s s3 bucket (s3://ornl-cumulus-prod-protected/above/ABoVE_LVIS_VegetationStructure/data) using NASA EarthData’s Common Metadata Repository. The data provide a comprehensive reference of boreal forest and Arctic tundra structure across a broad geographic extent and vegetation gradient, and can be used for understanding the uncertainty of structure estimates from spaceborne platforms. This data record contains 164,450 data files. There are 164,448 data files in GeoTIFF (*.tif) format, one file in geopackage (*.gpkg) format, and an R script file. It is also used in this Data Descriptor paper to map (Fig. 1) and visualize LVIS LiDAR data (Fig. 3), and summarize (Table 3) the LVIS acquisitions.

### LVIS Camera Imagery (ABOLVIS1A and OLVIS1A)

The optical camera imagery data records collected in 2017^27^ and 2019^28^ from the LVIS Camera-1 and Camera-2 systems, in JPEG format, are accessible through the NSIDC DAAC. This camera imagery, available in Level-1A, is geotagged (using a single geolocated reference point) and coincident to the LVIS LiDAR data records. This imagery includes nadir images of all overflown terrain, such as forests, tundra, lakes, and glaciers. It could be georeferenced and used to produce stereo digital elevations at high resolution, with nominal overlaps of 75% in the 2017 data record, and in 2019 of 67% (LVIS Camera-1) and 80% (LVIS Camera-2, Table 2). This optical camera imagery is also used to interpret results from the LiDAR collection. Additional details regarding the LVIS camera data records are available in Fig. 2 and Tables 2, 6.

### Naming conventions

LVIS LiDAR Level-1B and Level-2 data records, and Level-1A camera imagery from 2017 and 2019 generally use the same naming conventions. All Data Record IDs pertinent to the ABoVE campaign can be found in Table 6, and file naming conventions for the Level-1A, Level-1B and Level-2 data records are found in Tables 8–11. A major difference in the naming conventions occurs between the 2017 and 2019 deployments, as the LVIS platform began operating as a NASA Facility in 2018. Additional details about the LVIS file naming conventions can be found in the user guides for each LVIS data record, and the LVIS Technical Reference Document archived at the NSIDC DAAC^55^ and linked to all relevant data record landing pages.

**Table 8:** File Naming Convention for LVIS LiDAR data records from 2017 curated by the NSIDC DAAC (Level-1B and Level-2 data records) with the file naming convention: [Dataset ID]_ABoVE2017_MMDD_RYYMM_nnnnnn.NN.

Variable	Description
Dataset ID: LVIS1B, LVIS2	LVIS L1B = Short name for LVIS L1B Geolocated Return Energy Waveforms LVIS2 = Short name for LVIS L2 Geolocated Surface Elevation Product
ABoVE2017	Campaign identifier. ABoVE = acronym for Arctic-Boreal Vulnerability Experiment; 2017 = four-digit year of campaign
MMDD	Two digit month, two-digit day of start of data collection
RYYMM	Date (two-digit year, two-digit month) of data production
nnnnnn	Number of seconds since UTC midnight of the day on which data collection started
NN	Indicates file type: For LVIS1B:.h5 (HDF5 file),.h5.xml (XML metadata file); For LVIS2:.TXT (ASCII text file) or.TXT.xml (XML metadata file)

**Table 9:** File Naming Convention for LVIS camera imagery (Level-1A) from 2017 with the file naming convention: ABOLVIS1A_ABoVE2017_MMDD_RYYMM_nnnnnn.ext.

Variable	Description
ABOLVIS1A	LVIS L1A Geotagged Images
ABoVE2017	Campaign identifier: ABoVE = acronym for Arctic-Boreal Vulnerability Experiment; 2017 = four-digit year of campaign
MMDD	Two-digit month, two-digit day of start of data collection
RYYMM	Date (two-digit year, two-digit month) of data production
nnnnnn	Number of seconds since UTC midnight of the day on which data collection started
ext	File type:.JPG (JPG data file) or.JPG.xml (XML metadata file)

**Table 10:** File Naming Convention for LVIS Classic and Facility LiDAR data records from 2019 curated by the NSIDC DAAC (Level-1B and Level-2 data records) with the file naming convention: [Dataset ID]_ABoVE2019_MMDD_RYYMM_nnnnnn.ext.

Variable	Description
Dataset ID: LVISC1B LVISC2 LVISF1B LVISF2	LVISC1B - LVIS Classic Level-1B Geolocated Return Energy Waveforms LVISC2 - LVIS Classic Level-2 Geolocated Surface Elevation and Canopy Height Product LVISF1B - LVIS Facility Level-1B Geolocated Return Energy Waveforms LVISF2 - LVIS Facility Level-2 Geolocated Surface Elevation and Canopy Height Product
ABoVE	Campaign identifier: ABoVE is the acronym for the Arctic-Boreal Vulnerability Experiment
2019	Four-digit year of campaign
MMDD	Two-digit month, two-digit day of start of data collection
RYYMM	Date (two-digit year, two-digit month) of data production
nnnnnn	Number of seconds since UTC midnight of the day on which data collection started
ext	File type/extension: LVISC1B and LVISF1B:.h5 (HDF5 data file) or.h5.xml (XML metadata file) LVISC2 and LVISF2:.txt (ASCII text data file) or txt.xml (XML metadata file)

**Table 11:** File Naming Convention for LVIS camera imagery (Level-1A) from 2019 with the file naming convention: OLVIS1A_CAMERA_ABoVE2019_MMDD_RYYMM_nnnnnn.ext.

Variable	Description
OLVIS1A	LVIS Level-1A Geotagged Images, Version 1 data record
CAMERA	Indicates the camera system used: CAM1 or CAM2 = Canon EOS 5DS R or Canon EOS 5D Mk II (see Table 2 or the LVIS Technical Reference^55^ for specific model and lens)
ABoVE2019	Campaign identifier or primary location. ABoVE = Arctic-Boreal Vulnerability Experiment; 2019 = four-digit year of campaign
MMDD	Two-digit month and two-digit day of start of data collection
RYYMM	Date (two-digit year, two-digit month) of data production
nnnnnn	Number of seconds since GPS midnight of the day the data collection started (Canon only)
ext	File type:.JPG (JPEG data file) or.CR2 (Canon Raw Version 2 data file),.JPG.xml (XML metadata file)

The LVIS ABoVE L3 data record has a slightly different naming convention than the other LVIS data records (see Table 12), as it was created as a gridded data record following the archival of the Level-2 data records.

**Table 12:** File Naming Convention for the ABoVE LVIS Level-3 data record (based on Level-2 Facility-only data records) curated by the ORNL DAAC with naming convention: LVISF3_ABoVEYYYY_MMDD_FLIGHTLINE_GRIDNAME_STAT_30 m.tif.

Variable	Description
YYYY	Year of collection (2017 or 2019)
MMDD	Date (two-digit year, two-digit month) of data production
FLIGHTLINE	Flight line number
GRIDNAME	Variable name as developed for the Level-3 data record
STAT	Mean, maximum, minimum, or count described for the specific variable of the Level-3 data record
30m	Spatial resolution of the data record
.tif	File type: GeoTiff

## Technical Validation

### LVIS Data from the NASA Goddard Team - Level-1A, 1B and Level-2

The LVIS team at NASA Goddard has rigorously verified the accuracy of the different LVIS data records. For the 2019 deployment, engineering check flights were flown over sites in Maryland, Virginia, and North Carolina on 7 November 2018 and 31 January 2019 out of NASA’s Langley Research Center in Hampton, VA to validate the LVIS instrument^55^. Validation data from these test flights were consistent with past research in dense tropical forests studied by the LVIS team which showed LVIS elevations to be within 1.5 m of coincident *in situ* ground elevation data on slopes less than 3°, and within 5 m of each other (on slopes of up to 30°)^14,20,54^. As mentioned in the Methods section, laser positioning at the time of each laser shot was provided by GPS satellite data, with laser pointing information provided by an IMU attached directly to the LVIS instrument. Data were also compared with a digital elevation model (TanDEM-X 90 m DEM)^86^ to check for outlier returns.

### LVIS Camera Imagery - Level-1A

Assessments by the LVIS team at NASA Goddard have shown no known errors or limitations in the 2017 LVIS Level-1A camera imagery.

For the 2019 LVIS Level-1A camera imagery, a known issue with the camera imagery is the potential for a 1-second offset in the image collection time contained in the Exif “GPS Date/Time” field^55^. For these images, the last six numbers in the file name refer to the time when the image was taken (showing the number of seconds past GPS midnight on the day data collection started). For images noted in Table 13, the collection time contained in the file name should be used, or one second should be added to the time contained in the Exif “GPS/Time” field.

**Table 13:** LVIS 2019 deployment camera imagery known issues include a 1-second offset in image collection time as located in the Exif “GPS Date/Time” field for some dates.

Date	Cameras affected
2019-07-12	Camera 1 and Camera 2
2019-07-13	None
2019-07-15	None
2019-07-16	None
2019-07-18	None
2019-07-22	Camera 1 and Camera 2
2019-07-23	Camera 1
2019-07-25	Camera 1 and Camera 2
2019-07-27	Camera 1 and Camera 2
2019-07-28	Camera 1 and Camera 2
2019-07-29	None
2019-07-31	Camera 1 and Camera 2
2019-08-01	None
2019-08-07	None

Refer to Table 2 for more information on these cameras.

### 2017 and 2019 LVIS LiDAR Level-1B: Classic and Facility

Lower quality data, such as data collected in areas with clouds and cloud-obscured returns, were removed based on comparison with a digital elevation model (TanDEM-X 90 m DEM)^86^; however, spurious returns may still be present. Data collected during aircraft turns have been removed from these data records. It is recommended that users review the waveforms for their specific areas of study to verify ground return and canopy top identification. It is possible that some anomalies are still present in the data and users are encouraged to use available tools such as the LVIS Product Data Viewer^87^ (described in the Usage Notes section below) to further investigate data in their areas of interest.

### 2017 and 2019 LVIS LiDAR Level-2: Classic and Facility

For the Level-2 data records, obvious lower quality data, such as data collected in areas with clouds and cloud-obscured returns, were removed based on comparison with a digital elevation model (TanDEM-X 90 m DEM)^86^; however, spurious returns may still be present. Atmospheric conditions (fog, haze, and blowing snow) in sections of flights can cause multiple scattering, which can appear as a tail in the return waveforms. Data collected during aircraft turns have been removed from these data records. It is recommended that users review the waveforms for their specific areas of study using available tools such as the LVIS Product Data Viewer^87^ (described in the Usage Notes section below) to verify surface return and canopy top identification.

Recent studies have used the LVISF2 data record in comparisons with drone- and satellite-based imagery. Van der Sluijs *et al*.^52^ used LVISF2 to assess vegetation canopy height modelling capabilities from beyond-visual-line-of-sight (BVLOS) aerial drones. Their comparisons showed satisfactory agreement for height metrics (r^2^ value of 0.7–0.8, RMSEs <1.0 m), with the highest correlations between the BVLOS canopy height model (<20 cm resolution) and LVISF2 for LVIS height estimates at the 95th and 99th height percentiles (RH95 and RH99). For satellite-based comparisons, Travers-Smith *et al*.^15^ used the LVISF2 data record to validate ICESat-2 derived canopy height predictions (30 m resolution), which showed a more moderate agreement (r^2^ value of 0.44, and an overall RMSE of 2.69 m). Similarly, Feng *et al*.^47^ assessed the accuracy of the ICESat-2 ATL08 product using LVISF2 and found strong agreement for segments of different resolutions: 20 m (RMSE = 4.17 m; bias = 0.08 m) and 100 m segments (RMSE = 4.75 m; bias = 0.88 m). The differing spatial resolutions between the drone- and satellite-based imagery and the LVISF2 data record offers one possible influence on the results of these comparisons.

### Level-3 LVIS LiDAR Data

The LVIS LiDAR Level-2 Facility data record coincides spatially with existing small-footprint discrete return airborne LiDAR from NASA Goddard’s Lidar, Hyperspectral, and Thermal (G-LiHT) Imager^88,89^, enabling direct comparison between the datasets^19^. When the gridded ABoVE LVIS L3^76^ data record was compared with the G-LiHT dataset regridded to matching 30 m resolution, LVIS captured the top of the canopy well with r^2^ values from 0.65–0.88, depending on observation year and tree canopy cover intervals ranging from 40 to 100%^19^. The relationships were weakest in the <20%, and up through 40%, tree canopy cover intervals, especially where there was a larger time difference (5 years) between the collections. Additionally, this Level-3 data record is now being used to validate a circumpolar map of boreal forest biomass^43^.

## Usage Notes

Numerous custom open source tools exist for utilizing LVIS data records. Members of the LVIS user community developed the LVIS Product Data Viewer^87^ (available on GitHub and archived through Zenodo) to interactively preview LVIS LiDAR Level-1B and Level-2 data records in their native formats (HDF5 and ASCII text files, respectively). Additionally, the NSIDC DAAC makes available a repository of LVIS tools, initially developed by the LVIS team at NASA Goddard Space Flight Center (nsidc/lvis-tools), and now available on Github^90^. The package requires a C compiler and IDL to run. While initially developed for a different LVIS deployment, users of the ABoVE LVIS data record collection may find this code useful. Lastly, the LVIS team at NASA Goddard Space Flight Center provides python code to plot waveform data and elevation, including locations of the ground modes^91^. Additional usage specifics for some of the data records can be found below.

### LVIS Level-1A Camera Imagery

The data files can be viewed using any software that recognizes the JPG format. Frame ID markers (requires Google Earth to view KMZ files) are available at the NASA LVIS-ABoVE campaign websites for each year of data collection^84,85^.

### LVIS Level-1B LiDAR Data

The following external tools provide access to software for reading and viewing HDF5 data files. Please be sure to review instructions on installing and running the programs.

- LVIS Product Data Viewer^87^ (described above).
- HDFView: Visual tool for browsing and editing HDF4 and HDF5 files.
- Panoply netCDF, HDF and GRIB Data Viewer: Cross-platform application. Plots geo-gridded arrays from netCDF, HDF and GRIB datasets.
- For additional tools, see the HDF-EOS Tools and Information Center.
- Also available: read_ilvis1b.pro, an IDL program that reads the LVIS Level-1B data into an IDL structure^90^.

### LVIS Level-2 LiDAR Data

The data files can be opened by any software that reads ASCII text files. Also available: read_ilvis2.pro, an IDL program that reads the LVIS Level-2 data into an IDL structure^90^, the LVIS Product Data Viewer^87^ (described above), and sample R code (LVISF2-R-processing, on GitHub)^92^ developed by others in the LVIS community which can take LVISF2 ASCII files and write height information to rasters with desired spatial resolution and projection.

## Data Availability

All data records described within this research have been published and archived at a NASA archive center: LVIS LiDAR data Level-1B^78–80^ and Level-2^24,56,81^ data records, and LVIS Level-1A camera imagery^27,28^ can be found through the NSIDC DAAC; the LVIS Level-3 gridded LiDAR data record^76^ is available through the ORNL DAAC. All NASA Earth science data records are now being made available in NASA Earthdata^46^ and more information is available in the Data Records section. Table 6 provides a summary of the collection of available data records, along with their formats and repository locations.

## Code availability

Code referenced in the Usage Notes section is publicly accessible at the specified repositories: the LVIS Product Data Viewer^87^ (10.5281/zenodo.16334757) is available via Zenodo and GitHub, NSIDC LVIS tools^90^ (https://github.com/nsidc/lvis-tools) via GitHub, and the LVISF2-R-processing^92^ sample code (https://github.com/hztraver/LVISF2-R-processing) via GitHub. The NASA Goddard Space Flight Center LVIS team tools are available via their website^91^ (https://lvis.gsfc.nasa.gov/CodeUtilities/utilities_home.html).
